# Dysbiosis of the Human Oral Microbiome During the Menstrual Cycle and Vulnerability to the External Exposures of Smoking and Dietary Sugar

**DOI:** 10.3389/fcimb.2021.625229

**Published:** 2021-03-19

**Authors:** Nagihan Bostanci, Maria Christine Krog, Luisa W. Hugerth, Zahra Bashir, Emma Fransson, Fredrik Boulund, Georgios N. Belibasakis, Kristin Wannerberger, Lars Engstrand, Henriette Svarre Nielsen, Ina Schuppe-Koistinen

**Affiliations:** ^1^Division of Oral Diseases, Department of Dental Medicine, Karolinska Institutet, Stockholm, Sweden; ^2^The Recurrent Pregnancy Loss Units, Copenhagen University Hospitals, Rigshospitalet and Hvidovre Hospital, Copenhagen, Denmark; ^3^Department of Clinical Immunology, Copenhagen University Hospital, Rigshospitalet, Denmark; ^4^Centre for Translational Microbiome Research, Department of Microbiology, Tumor and Cell Biology, Karolinska Institutet, Stockholm, Sweden; ^5^Science for Life Laboratory, Stockholm, Sweden; ^6^Department of Obstetrics and Gynaecology, Holbæk Hospital, Holbæk, Denmark; ^7^Ferring International Center SA, Saint-Prex, Switzerland; ^8^Department of Obstetrics and Gynaecology, Hvidovre Hospital, Copenhagen, Denmark; ^9^Department of Clinical Medicine, University of Copenhagen, Copenhagen, Denmark

**Keywords:** menstrual cycle, oral microbiome, saliva, hormonal contraceptives, sugar, diet, women’s health, shotgun sequencing

## Abstract

Physiological hormonal fluctuations exert endogenous pressures on the structure and function of the human microbiome. As such, the menstrual cycle may selectively disrupt the homeostasis of the resident oral microbiome, thus compromising oral health. Hence, the aim of the present study was to structurally and functionally profile the salivary microbiome of 103 women in reproductive age with regular menstrual cycle, while evaluating the modifying influences of hormonal contraceptives, sex hormones, diet, and smoking. Whole saliva was sampled during the menstrual, follicular, and luteal phases (n = 309) of the cycle, and the participants reported questionnaire-based data concerning their life habits and oral or systemic health. No significant differences in alpha-diversity or phase-specific clustering of the overall microbiome were observed. Nevertheless, the salivary abundances of genera *Campylobacter*, *Haemophilus*, *Prevotella*, and *Oribacterium* varied throughout the cycle, and a higher species-richness was observed during the luteal phase. While the overall community structure maintained relatively intact, its functional properties were drastically affected. In particular, 11 functional modules were differentially abundant throughout the menstrual cycle, including pentose phosphate metabolism, and biosynthesis of cobalamin and neurotransmitter gamma-aminobutyric acid. The menstrual cycle phase, but not oral contraceptive usage, was accountable for greater variations in the metabolic pathways of the salivary microbiome. Further co-risk factor analysis demonstrated that *Prevotella* and *Veillonella* were increased in current smokers, whereas high dietary sugar consumption modified the richness and diversity of the microbiome during the cycle. This is the first large study to systematically address dysbiotic variations of the oral microbiome during the course of menstrual cycle, and document the additive effect of smoking and sugar consumption as environmental risk factors. It reveals the structural resilience and functional adaptability of the oral microbiome to the endogenous hormonal pressures of the menstrual cycle, while revealing its vulnerability to the exogenous exposures of diet and smoking.

## Introduction

The oral cavity is a special ecological habitat composed of soft and non-shedding hard tissues, colonized with a plethora of microorganisms (Wade, 2013; Belibasakis et al., 2019). The oral microbiota constitutes the second most diverse microbial community of the human body, which under normal circumstances remains mostly stable (Human Microbiome Project, 2012; David et al., 2014; Rosier et al., 2018; Lif Holgerson et al., 2020). The continuous presence of saliva, a unique biological medium, has a crucial role in maintaining the stability of the oral microbiota. Higher microbial diversity in saliva, with altered pH and proteolytic enzyme activity was proposed to indicate early dysbiosis towards inflammatory oral diseases (Zaura et al., 2009). Salivary composition and flow rate vary with age, sex, daily rhythm, dietary habits as well as female hormones (Yeh et al., 1998; Lukacs and Largaespada, 2006; Gumus et al., 2015). Female hormones, specifically estrogens, may suppress the physiological salivary flow rate (Streckfus et al., 1998; Lu et al., 1999). This results in reduction of the natural antimicrobial capacity of saliva, thus disrupting the local microbial homeostasis and increasing susceptibility to gingivitis and dental caries in the affected women (Lukacs and Largaespada, 2006; Gursoy et al., 2008; Silva de Araujo Figueiredo et al., 2017).

The reproductive age of a woman’s lifetime, spanning from puberty until menopause is characterized by major changes in circulating female hormone levels and frequently accompanied by emotional and physiological changes, including heightened inflammatory status (Oertelt-Prigione, 2012; Clancy et al., 2013). The salivary and plasma kinetics of sex steroids mirror one-another across the menstrual cycle (Gandara et al., 2007). Of note, salivary estradiol levels peak at the time of ovulation, aligning with compositional changes of uterine endocervical gland secretions (Saibaba et al., 2017). Among the most common oral signs observed during menstruation are gingival inflammation, reduced salivary flow, altered pH and oral ulcers (Holm-Pedersen and Loe, 1967). Furthermore, the use of hormonal contraceptives has been associated with poorer oral health in young women, yet the mechanistic links between such hormone supplementation and oral microbial dysbiosis is not elucidated (Brusca et al., 2010). Long‐term use of oral contraceptives can lead to accelerated progression of periodontal disease, yet this may also be concentration-dependent (Preshaw et al., 2001; Preshaw et al., 2013). Early studies performed shortly after the introduction of oral contraceptives, when hormone doses were very high, found evidence of increased gingival inflammation and possibly also increased probing depths, often despite better oral hygiene in the users of the oral contraceptives (Preshaw et al., 2001). Low doses of estrogen and progesterone are now being widely used in contraceptive pills, and these have little impact on the extent of periodontal inflammation in response to plaque accumulation (Preshaw et al., 2001). Most studies in the field are based on clinical parameters of disease severity and do not take into account direct measures of oral infection, such as qualitative and quantitative microbiome changes in the oral milieu (Holm-Pedersen and Loe, 1967). The few studies available on the dynamic interplay between menstrual cycle hormones or hormonal contraceptives and selected components of the oral microbiota did not reach a clear consensus (Jensen et al., 1981; Fischer et al., 2008; Kumar, 2013). Interestingly, ovulation also has been linked to the increased levels of anaerobic bacterial counts in saliva independent of flow rate (Prout and Hopps, 1970). Jensen et al. reported that women who are taking oral contraceptives had up to sixteen times higher level of *Bacteroides* species in their dental plaque than the control group (Jensen et al., 1981).

Data regarding the dynamics of the salivary microbiome during the regular menstrual cycle using high-throughput sequencing technologies in young women is entirely missing. Therefore, the aim of the present study was to examine the structural and functional dynamics of the salivary microbiome during one full menstrual cycle in women of reproductive age under different contraceptive regimens.

## Materials and Methods

### Study Design and Ethics Statement

Women were recruited by advertisements in student magazines, university notice boards, and social media and included between September 2017 and January 2018 at Rigshospitalet, Copenhagen, Denmark. All data were collected and managed using REDCap electronic data capture tools, hosted at the Capital Region of Denmark. The study is approved by The Regional Committee on Health Research Ethics (H-17017580) and the Data Protection Agency in the Capital Region of Denmark (2012-58-0004). All participants gave oral and written consent to participate. All participants were asked to phone the clinic when spotting/beginning of bleeding. They were then scheduled for a hospital visit on cycle day 1–3. An ultrasound scan was performed to confirm that the participant was not pregnant and to confirm the menstruation with shedding of the endometrium. Additionally, hormone levels (plasma estradiol and progesterone) were measured. The two following visits were booked (day 8–12) and (day 18–22) every time with an ultrasound scan to confirm cycle phase and absence of pregnancy. One of the inclusion criteria was to have a regular cycle (median 26 days). The subjects were divided into 3 groups: group 1 (n = 43) that did not use any hormonal contraceptives, group 2 (n = 41) that used combined oral contraception (COC: estrogen + progestin) for at least half a year prior to the start of the study and group 3 (n = 19) using levonegestrel intra-uterine system (LNG-IUS). Women were excluded from the study if they were pregnant or had an intention to become pregnant during the course of the study or had oligomenorrhea or irregular menstrual cycles or spotting. Subjects were also excluded if they had any systemic disease or medical condition or been treated by antibiotics in the past three months prior to the beginning of the study or during its duration.

### Variables Reported by the Subjects Through Questionnaires

Participants reported data concerning their life habits and health history. The food frequency questionnaire was based on four-week recall, with frequencies given on a 9-point scale from “0 times in the past four weeks” to “>3 times/day for the past four weeks”. Frequency of free-sugar consumption was derived from the sum of the frequencies for the following food items: chocolate milk, juice, soda with sugar, ice-cream, biscuits and cookies, sweet bread and rolls, dry cake, cake with filling and candy (including chocolate, licorice, jelly and other candy). On quantitative analyses, sugar consumption was divided into low (up to and including the first quartile), high (above the third quartile), or intermediate. In relation to oral health, participants were asked whether they had been to a dentist or dental hygienist during the 3 months prior to answering the questionnaire. Smoking was coded as “daily smoker”, “occasional smoker”, “former smoker”, and “never smoker”.

### Blood and Saliva Collection and Processing

Women were followed during a full menstrual cycle including three hospital visits. The first hospital visit was at cycle day (CD) 1–3. The second visit was CD 8–12 and the third CD 18–22. The patients were fasting 30 min before saliva collection, including drinking, chewing gum or chewing tobacco, and smoking. Whole saliva samples (2 ml) were collected using a SalivaGene Collector (STRATEC Molecular GmbH, Germany) containing lyophilized DNA stabilization buffer, according to the instructions of the manufacturer, and were frozen at -80°C. Blood samples were drawn at every hospital visit. Blood was collected in 9 ml EDTA tubes, left until separated and spinned for 15 min at 3,000 rpm and plasma was aliquoted and frozen at -80°C. Plasma estradiol and progesterone were measured using the standard automated system (Cobas^®^ 8000 by Roche Diagnostics).

### Extraction of Salivary DNA and Next-Generation Sequencing

Saliva aliquots of 600 µl were shipped to CoreBiome (OraSure, Bethlehem, PA, USA) where they were extracted with MO Bio PowerFecal (Qiagen, Hilden, Germany) automated for high throughput on QiaCube (Qiagen), with bead-beating in 0.1 mm glass bead plates. Three spaced negative controls and one positive control were included in each extraction. All negative extraction controls had undetectable amounts of DNA, and all positive controls were also approved. DNA concentration (for samples and controls) was quantified using Quant-iT Picogreen dsDNA Assay (Invitrogen, ThermoFisher Scientific, Carlsbad, CA, USA). Libraries were prepared using an adapted Nextera (Illumina Inc, San Diego, CA, USA) procedure and sequenced on an Illumina NextSeq using single-end 150 bp reads with a NextSeq 500/550 High Output v2 kit. Reads were processed with CoreBiome’s BoosterShot shallow shotgun sequencing technology. The raw sequencing reads are available from the European Nucleotide Archive under project PRJEB37731, samples SAMEA6662389-SAMEA6662857.

### Bioinformatics Analysis

Human reads were removed by mapping to the hg19 release of the human genome using BBTools (available at https://sourceforge.net/projects/bbmap/). Because BoosterShot technology is optimized for fecal samples, taxonomy was reannotated the reads using Kraken2 (26) with confidence set to 0.5 and Bracken, based on the Human Oral Microbiome Database v9.0.3 (27). Functional annotation based on KEGG modules was used as provided by CoreBiome.

### Statistical Analyses

All statistical analyses were performed in R v. 3.5.2. Alpha-diversity was calculated as the observed number of species as well as Simpson’s inverted index. Comparisons for the same individual across time were calculated as paired t-tests, while comparisons between individuals were calculated using Welch’s t-test. Differences between the three contraception groups were calculated with Pearson’s chi-square test or Fishers Exact test for count data and Kruskal-Wallis test for continuous data. Differences in variation were quantified using Levene’s test of equality of variance, using the Brown-Forsythe variant with R package lawsat (v3.2). Correlations were calculated with Pearson’s product moment. All tests were performed with a 95% confidence interval and a significance cutoff of 0.05. Multiple testing correction was conducted with the Benjamini-Hochberg procedure where applicable. Beta-diversity was calculated on Bray-Curtis distances and clustered on complete linkage. The relative impact of metadata factors on beta-diversity dispersion assessed through Permanova. Alpha- and Beta-diversity analyses were calculated with package Vegan (v2.5-3) and graphs were generated with packages RColorBrewer (v1.1-2) and Vioplot (v0.2). Associations between specific taxa and the metadata were calculated in Maaslin2, treating the contraceptive and phase of the cycle as fixed effects and individual’s identities, smoking, and sugar intake as random effects. A minimum abundance of 0.1% in at least six samples was required to keep a taxon in the analysis. Furthermore, clustering of species was done according to the “color complex” classification of Socransky et al., (1998). (i.e., six-color cluster-lists of bacteria according to their frequency of detection and levels in periodontitis and health) and according to the core microbiome classification data from Abusleme et al. (health-associated core species and periodontitis-associated core species in the subgingival microbiome) (Abusleme et al., 2013). Correlations between color groups and metadata were calculated initially as multivariate ANOVA, and where differences were found, investigated as beta-regressions treating contraceptive, phase of the cycle, smoking and sugar intake as fixed effects and individual’s identities as random effects, using R package glmmTMB (v0.2.2.0).

## Results

### Demographic and Clinical Characteristics of the Study Participants

Demographic and clinical characteristics of the 103 study participants are categorized based on contraceptive use and summarized in **Table 1**. The participants reported normal menstrual cycles of approximately 26 days (range, 23 to 34 days). There were no significant differences (p > 0.05) in the age, BMI or frequency of tobacco or cannabis usage between the women in each contraceptive group (**Table 1**). For participants using COC, 25 of 41 reported a daily dosage of 150 µg levonorgestrel and 30 µg ethinylestradiol. The other participants had several different progestins, combined with a daily dosage of ethinylestradiol of 20–35 µg. A single participant was on a multiphasic pill, while three could not give the name of their pill. For participants with an IUS, three brand names were reported (Jaydess, Kyleena and Mirena, all produced by Bayer AB, Leverkusen, Germany), with four participants unable to name the brand of their device.

**Table 1:** Demographic and clinical characteristics of the study participants.

	Non-hormonal contraception n = 43	Combined oral contraception n = 41	Levonorgestrel intra-uterine system n = 19	p-value
**Age, years** **(median, IQR)**	23.0 (22.0–28.0)	23.0 (22.0–24.0)	24.0 (22.0–25.0)	0.452^1^
**BMI, kg/m^2^** **(median, IQR)**	21.8 (20.8–24.6)	22.5 (20.7–24.3)	21.6 (21.6–23.1)	0.725^1^
**Tobacco smoking (n)**	16	8	7	0.163^2^
Tobacco, Snus* (n)	4	5	0	0.316^3^
**Cannabis smoking (n)**	5	0	3	0.025^3^

IQR, interquartile range; BMI, Body Mass Index.

^1^Kruskall-Wallis test ^2^Chi-square test ^3^Fisher’s Exact test.

*Snus is a form of moist powder smokeless tobacco product.

A few of the participants had been to a dental health professional during the three months prior to the beginning of the study, with no difference between groups (**Supplementary Table 1**). Self-rated health was overall high and not significantly different between groups (**Supplementary Table 1**). Sugar consumption did not vary throughout the menstrual cycle or between the contraceptive groups (**Supplementary Table 1**).

### Microbial Community Structure and Composition

**Figure 1:**
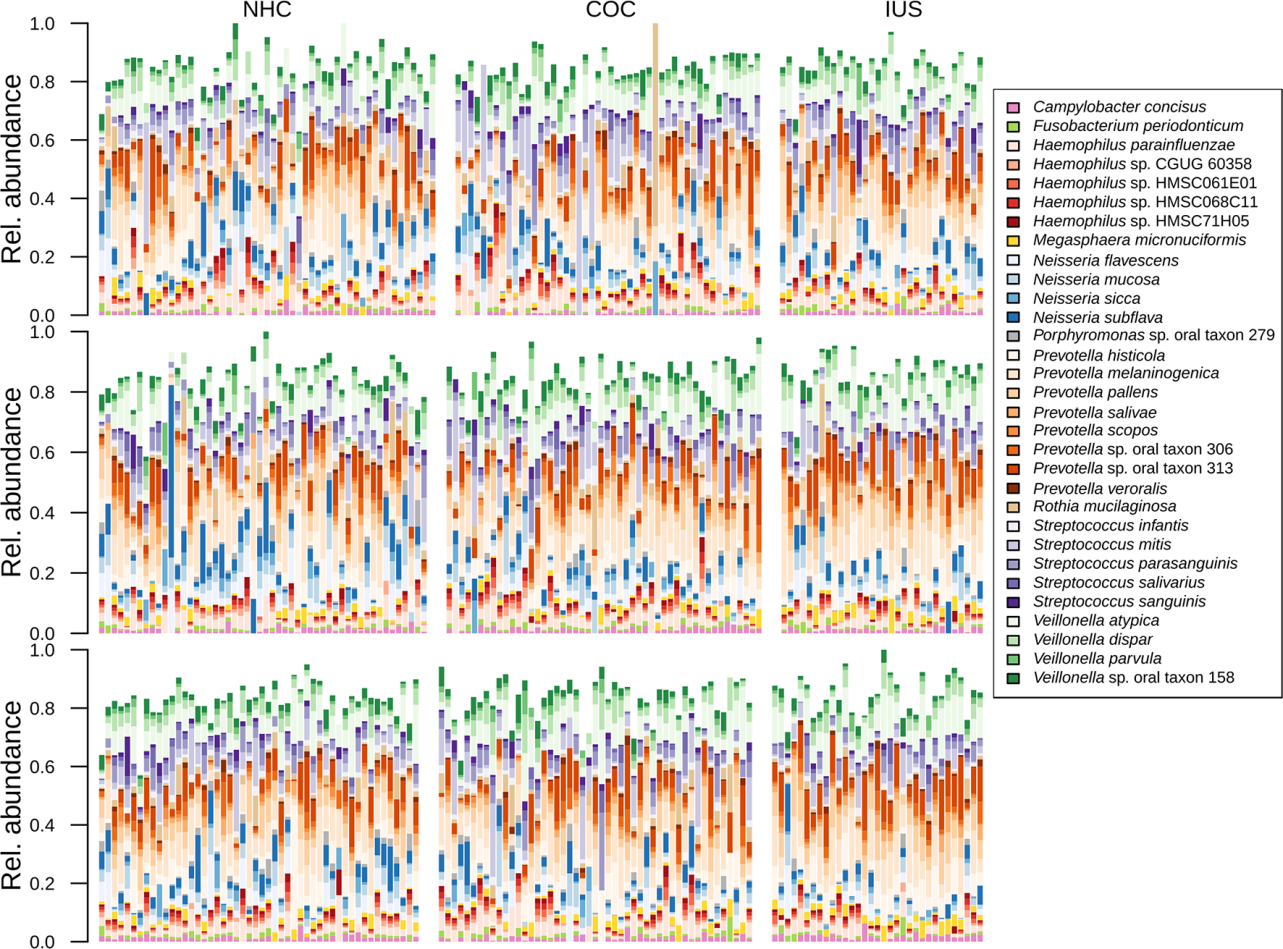
Bar plot displaying the taxonomic composition of samples, sorted by phase of the menstrual cycle and contraceptive method.

The total number of annotated sequence reads for the overall cohort of 103 women was 102,605,212 (median 242,834 reads per sample, IQR 106,839–482,885). There were 209 microbial taxa identified in the saliva samples. The taxonomic classification is presented in **Supplementary Table 2**. In brief, the OTUs were collectively represented by eight bacterial phyla, namely Actinobacteria (0–70%, median 4%), Bacteroidetes (0–66%, median 37%), Firmicutes (0–77%, median 31%), Proteobacteria (0–65%, median 20%), Fusobacteria (0–8.6%), Spirochaetes (0–1.4%), and candidate divisions *SR1* (0–0.4%) and TM7 (0–14%). Fifty genera were identified, of which the most abundant genera across all samples were *Haemophilus* (0–48%, median 7%, specially *H. parainfluenzae*, 0–23%)*, Neisseria* (0–53%, median 8%, specially *N. flavescens*, 0–21% and *N. subflava*, 0–23%)*, Prevotella* (0–65%, median 34%, specially *P. histicola*, 0–24%, *P. melaninogenica*, 0–26%, *P. pallens*, 0–20% and oral taxon 313, 0–25%)*, Streptococcus* (0–60%, median 13%, specially *S. mitis*, 0–60% and *S. parasanguinis*, 0–27%) and *Veillonella* (0–42%, median 11%, specially *V. atypica*, 0–29%) (**Figure 1**). In addition to these genera, a few samples presented with high levels of *Rothia mucilaginosa* (0–70%, median 1%) and *R. dentocariosa* (0–26%, median 0.2%).

To assess the relative effects of several metadata parameters on the structure of the microbiome, we ran a permutational analysis of variance (Permanova) including contraceptive usage, phase of the menstrual cycle, smoking status, free-sugar consumption, and individual identity. All factors are partially explanatory of the distance between samples (**Table 2**). While unspecific individual factors are dominating, contraception and cycle phase play a comparable role to well-known determinants of oral health, such as free-sugar consumption and smoking (**Table 2**). Therefore, the remaining analyses were adjusted for these factors when possible.

**Table 2:** Result of permutational analysis of variance shows the relative effect of important factors on differentiating the microbiome between samples.

Factor	Degrees of freedom	R²	p-value
**Smoking**	3	0.083	0.001
**Sugar consumption**	2	0.024	0.001
**Contraception**	2	0.019	0.001
**Cycle phase**	2	0.012	0.001
**Residuals**	290	0.862	NA

**Figure 2:**
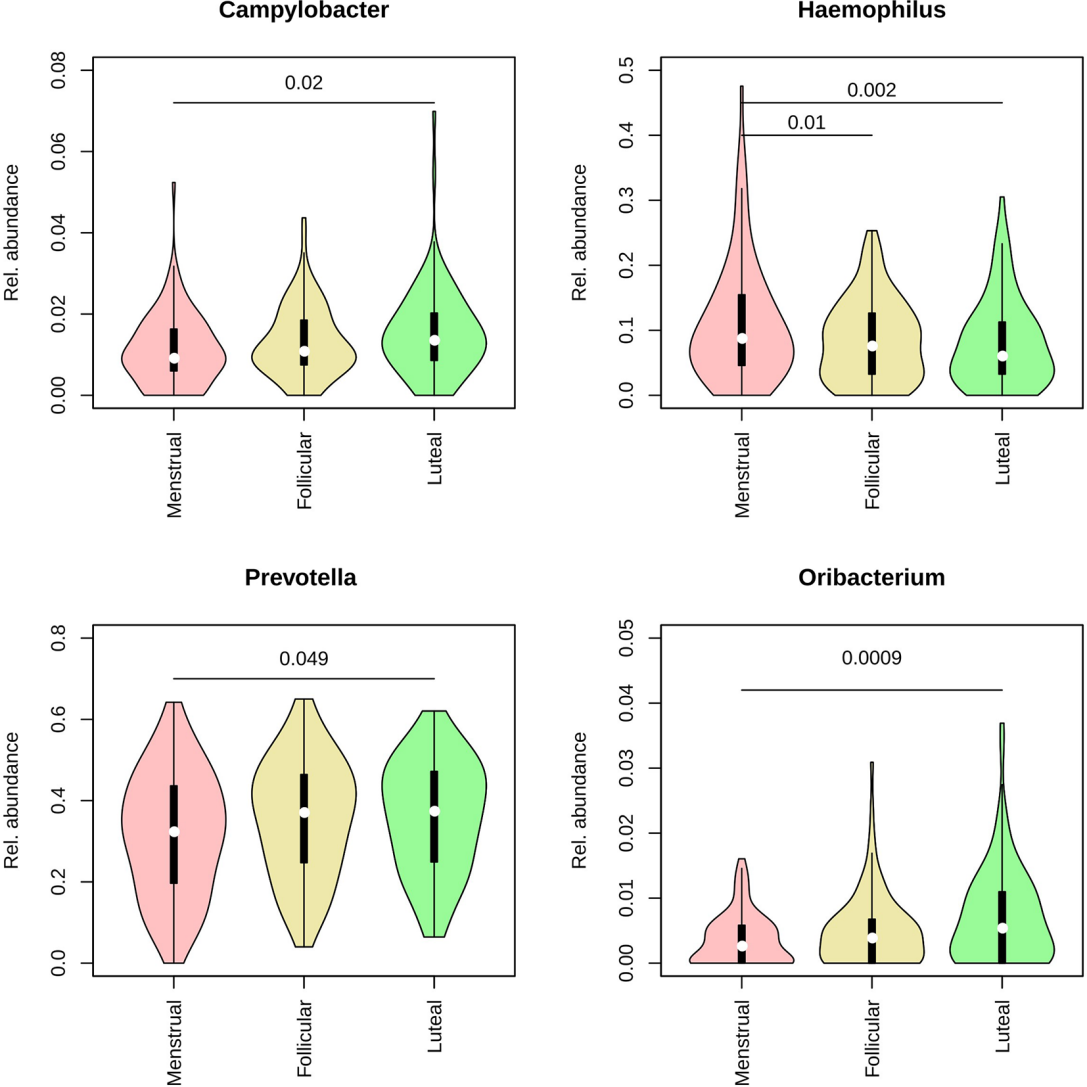
Violin plots representing the distribution in relative abundance for each phase of the menstrual cycle for the four genera found to vary across the cycle. Pink: menstrual. Yellow: follicular. Green: luteal.

To assess whether any specific taxa differed in abundance according to female hormonal cycles and contraception, we ran Maaslin2 using contraceptive and cycle phase as fixed effects and individual identity, smoking and sugar intake as random effects (see the methods section for details). We found that *Prevotella* and *Veillonella* were increased in current smokers (daily and occasional). Additionally, four genera were found to differ in abundance throughout the menstrual cycle, namely *Campylobacter*, *Haemophilus*, *Prevotella*, and *Oribacterium*, the latter of which was over-represented by the species *O. sinus* (**Figure 2**). In addition, the genus *Atopobium* was found to be more abundant in IUS users (r = 0.0017, p = 0.047).

### Oral Microbiome Richness and Diversity

**Figure 3:**
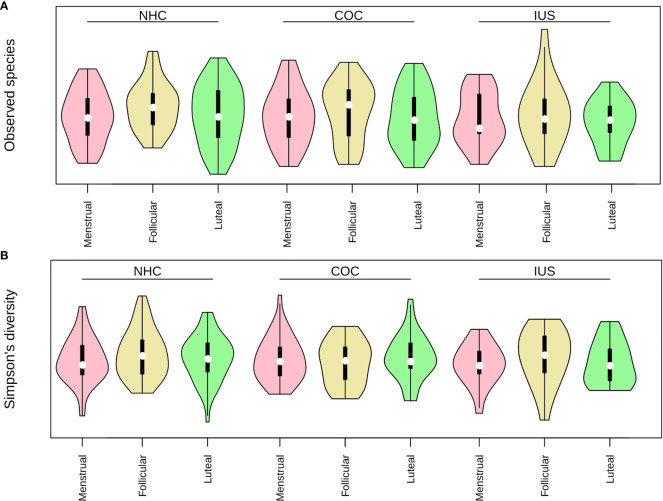
**(A)** Richness (observed species) and **(B)** Diversity (inverted Simpson's index) in each contraceptive group and phase of the menstrual cycle. Pink: menstrual. Yellow: follicular. Green: luteal.

**Figure 4:**
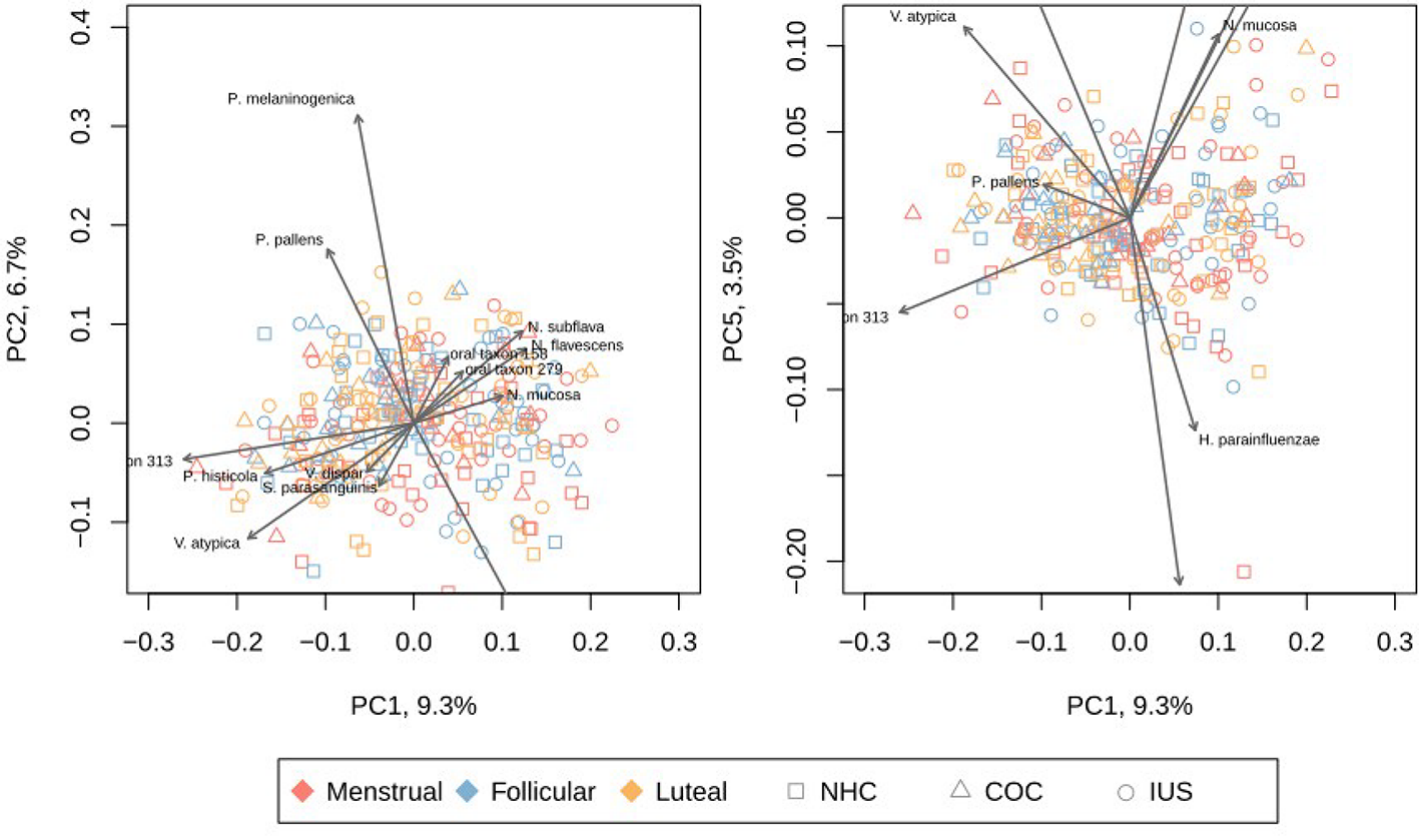
Principal Coordinates Analysis (PCoA) of each sample, based on Bray-Curtis distance. The left panel depicts PC1 and PC2, while the right panel depicts PC1 and PC5. Other principal components are depicted in **Supplementary Figure 3**. The species with highest impact on the depicted principal components are overlaid as grey arrows. Red: menstrual phase. Yellow: follicular phase. Blue: luteal phase. Circle: non-hormonal contraceptives. Square: combined oral contraceptives. Triangle: intra-uterine levonegestrel system. H. parainfluenzae, Haemophilus parainfluenzae; N. flavescens, Neisseria flavescens; N. mucosa, Neisseria mucosa; N. subflava, Neisseria subflava; oral taxon 306, Prevotella sp. oral taxon 306; oral taxon 313, Prevotella sp. oral taxon 313; P. histicola, Prevotella histicola; P. melaninogenica, Prevotella melaninogenica; P. pallens, Prevotella pallens; R. dentocariosa, Rothia dentocariosa; R. mucilaginosa, Rothia mucilaginosa; S. mitis, Streptococcus mitis; S. parasanguinis, Streptococcus parasanguinis; S. salivarius, Streptococcus salivarius; V. atypica, Veillonella atypica; V. dispar, Veillonella dispar.

No significant differences in alpha-diversity (within-sample diversity) were observed as a result of smoking, although daily smokers had slightly higher richness (number of species) than never smokers (means 36 vs. 34, p = 0.06). Participants in the middle and high ranges of sugar consumption had higher richness and diversity than those with low sugar consumption, but this different was only significant for the middle range (mean richness: low 32.8, mid 39.5, high 38.0; mean diversity: low = 14.7, mid = 16.6, high = 15.7. p-values: richness 0.003, diversity 0.005). We next evaluated alpha-diversity (within-sample diversity) according to menstrual phases or contraceptive categories. No significant differences in alpha-diversity were observed across menstrual phases or between contraceptive methods. Higher richness (number of species) was observed in the luteal phase than in the menstrual phase (**Figure 3A**), but this did not prove to be significant by a narrow margin (p = 0.06), neither for the entire cohort nor for any of the contraceptive groups separately. There were no significant differences in diversity (Simpson’s inverted index; **Figure 3B**). Total DNA amounts in the samples did not correlate with either richness or diversity (**Supplementary Table 3**). While both contraceptive method and phase of the menstrual cycle were statistically correlated to beta-diversity (between samples; **Table 2**), there was no distinct pattern of clustering by either of these factors (**Figure 4**; **Supplementary Figure 1**).

The separation between samples was mainly driven by gradients in the abundances of a few species. A gradient was evident with high levels of *N. subflava, N. mucosa, N. flavescens, R. mucilagniosa* and *H. parainfluenzae* on one side, and high levels *P. histicola, V. atypica* and *Prevotella* taxon 313 marked the other side. A second gradient displayed *P. pallens* and *P. melaninogenica* on one extreme, and chiefly *S. mitis* on the other. Interestingly, *P. pallens* and *P. melaninogenica* are not related to smoking status. No linear combination of taxa covered a large amount of the variation, with the first two principal components covering only 16% of total variance. Only when including the first 12 principal components was 50% of the total variance covered (**Supplementary Figure 1**).

### Specific Taxa Clustering

**Figure 5:**
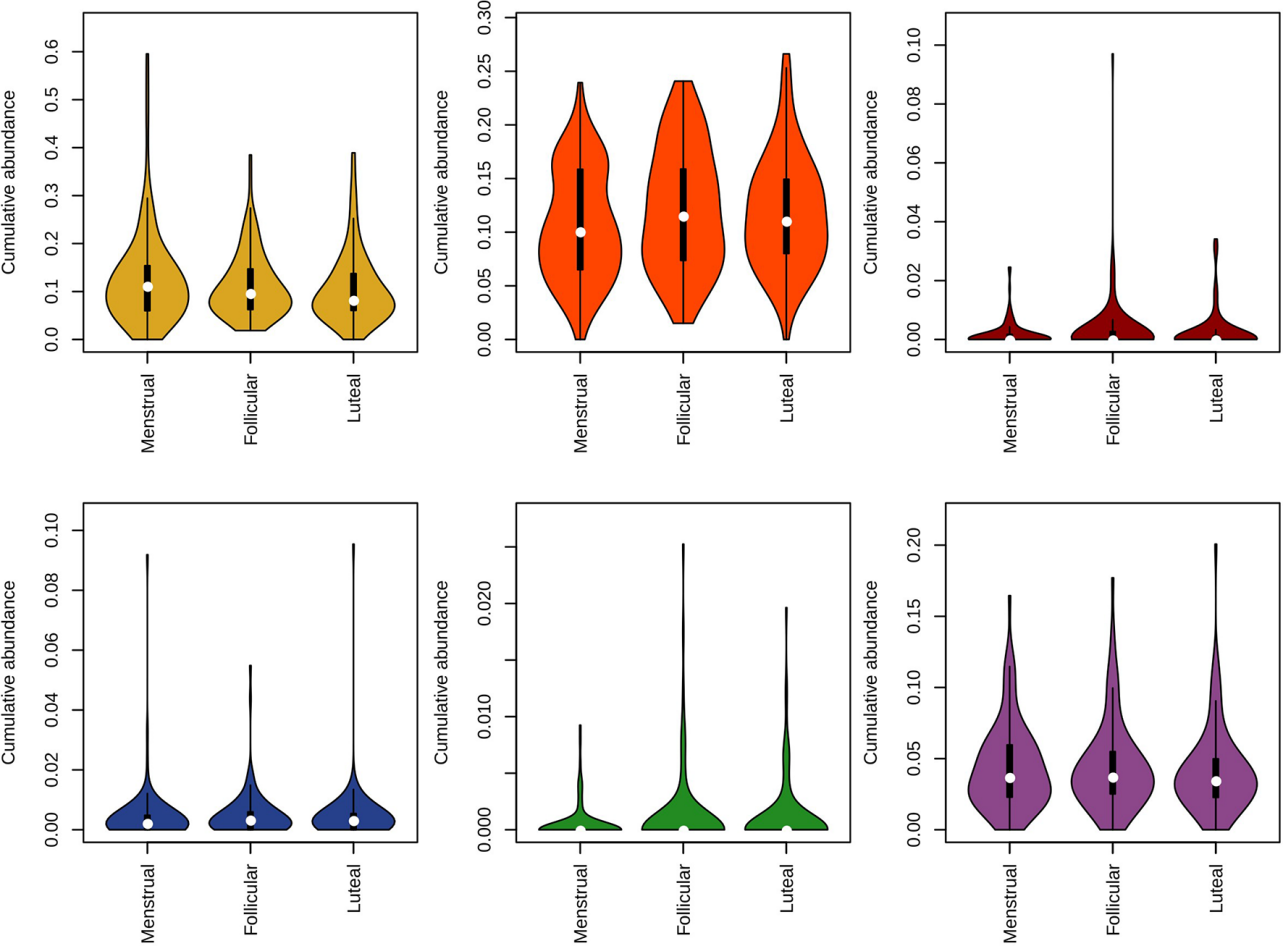
Violin plot representing the distribution in relative abundance for each phase of the menstrual cycle method for species of the Socransky complexes. The blue, yellow, green, and purple complexes are associated with periodontal health, whereas the orange and red complexes are correlated with periodontal disease.

Furthermore, clustering of species was done according to the “color complex” classification (6 “color complex”—blue, green, yellow, purple, orange, and red—based on their frequency of detection) and according to the core subgingival microbiome classification (health-associated core species and periodontitis-associated core species in the subgingival microbiome) (28, 29). The blue, yellow, green, and purple complexes are associated with periodontal health, whereas the orange and red complexes are correlated with periodontitis (Socransky et al., 1998). The bacteria in the metagenomic dataset were grouped into the color complexes. The percentages of each of these complexes per sample were analyzed. The most prevalent and abundant groups in this cohort were the yellow (present in 298/300 women, median abundance 9.5%), orange (297/300, 10.7%), and purple complexes (287/300, 3.7%) (**Figure 5**).

Multivariate analysis of variance (MANOVA) revealed the yellow group to be associated to contraception and sugar intake, while the purple group was associated to smoking and sugar consumption. These two groups were analyzed further with a beta-regression, adjusting for subject as a random factor. This analysis found the yellow group to be increased in high and intermediate sugar consumption (high: r = 0.31, p = 0.039; intermediate: r = 0.25, p = 0.05). The purple group was found to be decreased in the IUS group (r = -0.43, p = 0.016) and vary across smoking groups, being decreased among daily smokers (r = -0.57, p = 0.0055), increased among former smokers (r = 0.43, p = 0.0052) and not changed for occasional smokers (r = -0.06, p = 0.71). Furthermore, in relation to the menstrual cycle, we observed: a) a higher abundance of the yellow complex during the menstrual phase, b) a tendency for higher abundance of the red and green complexes during the follicular phase, and c) a lower abundance of the blue complex during the follicular phase (**Figure 5**). The interplay between each metadata factor and each of the colored groups is depicted as a heatmap in **Supplementary Figure 2**.

**Figure 6:**
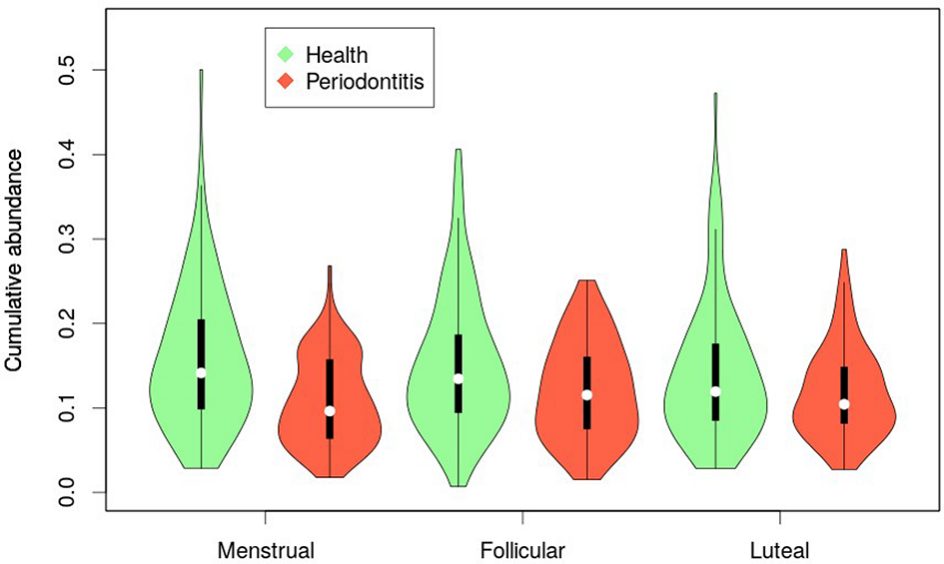
Violin plot representing the distribution in relative abundance for each phase of the menstrual cycle according to the subgingival core microbiome classification (green: health-associated; red: periodontitis-associated).

When classified according to the two core microbiome groupings (health- or disease-associated), there were also no notable differences in abundances between phases of the menstrual cycle (**Figure 6**), or between groups of contraceptives (**Supplementary Figure 3**). The variance in the abundance of health-associated bacteria within the COC group was more than twice greater than that of the other two groups (**Figure 6**; **Supplementary Figure 3**; COC, 0.012; NHC, 0.005; IUS 0.004; Levene’s test, p = 10^-4^). This difference was mostly driven by the dominance of the yellow complex, typically associated with periodontal health (**Figure 5**). Bacteria associated with caries were only found in low abundances (median 0, IQR 0–0.13%) and did not vary across the cycle (**Supplementary Table 4**). **Supplementary Table 4** lists all observed bacterial species and their association with health and disease.

### Shifts of Functional Gene Composition

**Figure 7:**
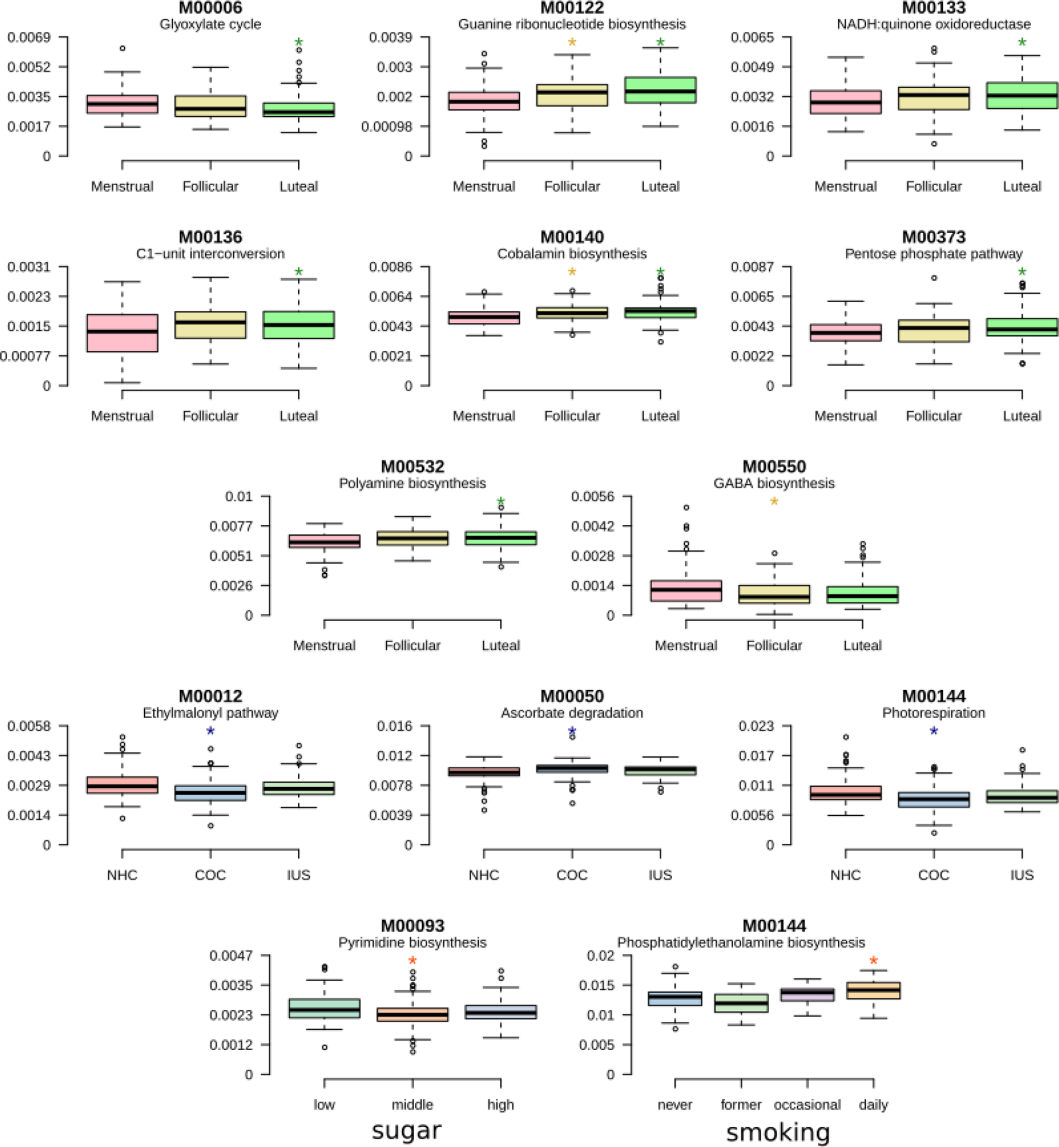
Shifts of functional gene composition. Boxplots presenting pathways that are differentially abundant during the menstrual cycle or according to contraceptive usage. Detailed results are presented in **Supplementary Table 5**.

To examine the differential representation of particular microbial metabolic and biosynthetic pathways during the menstrual cycle or with different contraceptive use, the functional potential of each sample was assessed based on KEGG modules. These were submitted to Maaslin2 with sugar intake, smoking, cycle phase, and contraceptive as fixed effects and the individual as a random effect, as was done for the taxonomic annotation. A single module, pyrimidine deoxyribonucleotide biosynthesis, was increased for daily smokers, and a single module, Phosphatidylethanolamine biosynthesis, decreased with sugar consumption. 11 modules were found to be differentially abundant over the menstrual cycle (**Figure 7**; **Supplementary Table 5**). Amongst these are pathways involving co-factors, such as cobalamin biosynthesis and ascorbate degradation, as well as the biosynthesis of the neurotransmitter *gamma*-aminobutyric acid (GABA). Another 3 modules varied with contraceptive usage, namely the glyoxylate cycle, guanine biosynthesis and the NADH:quinone oxidoreductase step of oxidative phosphorylation (**Figure 7**). Overall, functional analysis indicated that more variation is attributed to the phase of menstrual cycle rather than contraceptive usage.

### Hormonal Effects

Because standard hormonal measurements only measure endogenous estradiol and progesterone, not the ethinylestradiol, progestin and levonorgestrel used in COC and IUS, respectively, direct associations between female reproductive hormones and the oral microbiome were only conducted for women not using hormonal contraception (Stanczyk and Clarke, 2010). The estradiol levels were higher in the follicular and luteal phase compared to the menstrual phase (Mean ± STDEV: 0.51 ± 0.24, 0.41 ± 0.31, 0.14 ± 0.05 nmol/L, respectively) (**Supplementary Table 3**). The progesterone concentrations were increased by 19-fold in the luteal phase compared to follicular and menstrual phases (Mean ± SDEV: 32.10 ± 20.45, 1.53 ± 1.21, 1.67 ± 2.78 nmol/L).

The MANOVA indicates that the red group is dependent on estradiol levels. The beta-regression adjusted for sugar intake and smoking did not converge, but visual inspection of the scatter plot between the red group and estradiol levels reveals that, for subjects where red group bacteria are present, they increase with increasing estradiol (**Supplementary Figure 4**). Adjusting for smoking and sugar intake, 24 species were correlated to estradiol and another 30 to progesterone, but only a single bacterial species was found to be significantly correlated to estradiol after correction for multiple testing, namely, *Porphyromonas endodontalis* (r = 0.0018, adj. p = 0.039)

## Discussion

In the present study, we longitudinally characterized the salivary microbiome of 103 regularly menstruating women in reproductive age during the course of one full menstrual cycle, using whole genome (shotgun) sequencing, and evaluated its potential deviations caused by the use of oral contraceptives and changes during the menstrual cycle. Such fluctuations may render the oral microbiome more dysbiotic, able to drive more aberrant inflammatory responses by the gingival tissues, a well recognizable clinical feature at phases of the menstrual cycle. To the best of our knowledge, this is the first large study to systematically address dysbiotic variations of the oral microbiome during the course of menstrual cycle, and document the additive effect of smoking and sugar consumption as exogenous risk factors. The analysis of this cohort cumulatively identified 50 bacterial genera belonging to eight phyla, all well represented in the human oral microbiome database and previously identified as members of the human salivary microbiome (Dewhirst et al., 2010; Segata et al., 2012; Hasan et al., 2014).This diversity may be considered much lower compared to most salivary microbiome studies that have been using 16S rRNA gene sequencing instead, a method which may nevertheless overestimate diversity. Conversely, high species-specificity was notable here, with 209 species being detectable in the studied cohort. An earlier metagenomic survey of saliva reports that the salivary microbiome typically contains 175 bacterial species (Hasan et al., 2014). It estimated that the number of species-level phylotypes may vary from 500 to 10,000 and each oral niche may harbor on average 266 species level phylotypes (Zaura et al., 2009; Bik et al., 2010; Hasan et al., 2014). The lack of significant differences in taxa diversities across phases of the menstrual cycle, or use of contraceptives, denote the less susceptibility of the oral cavity to inherent biological or external pharmacological pressures, respectively (Zaura et al., 2014; Zaura et al., 2017). Dietary habits, including sugar intake, appeared to influence the composition of the salivary microbiome during the menstrual cycle, as evaluated by weekly dietary records. The finding that diet did influence the community composition of the salivary microbiome may not come as a surprise, as it has been shown that the influence of diet occurs also at the metabolome level (De Filippis et al., 2014; Tanner et al., 2018). Specific dietary habits i.e., high frequency of carbohydrate exposure can select for more acid-tolerant and acidogenic bacteria such as *Streptococci, Lactobacilli or Bifidobacteria*, which may in turn disturb the enamel mineral equilibrium, leading to irreversible demineralization and dental caries. Still, pairing metagenomic data with conventional nutrient profiles may not be sufficient to infer microbiome variations to diet (Johnson et al., 2019).

Despite the overall relative stabilities in diversity and overall abundance of the microbiome profiles in saliva, some taxon-function specific and significant changes were observed. In particular, four genera were found to differ in abundance throughout the menstrual cycle, namely *Campylobacter*, *Haemophilus*, *Prevotella*, and *Oribacterium*. Higher abundance of *Prevotella* species in saliva may increase pH and stimulate the flow of gingival crevicular fluid (Raber-Durlacher et al., 1994). These changes may favor acid-intolerant, proteolytic species associated with gingival inflammation. *P. intermedia* growth and biosynthetic activity appear to be regulated by progesterone and estradiol, two cycle hormones, as demonstrated in experimental models (Kornman and Loesche, 1982).

The salivary and plasma dynamics of sex steroids seems to mirror one-another across the menstrual cycle, with progesterone levels peaking during the luteal phase and estradiol levels during the follicular phase (Gandara et al., 2007). Further, the estradiol levels in plasma positively correlated with the total number of the bacteria from the red complex bacteria (*P. gingivalis*, *T. denticola*, *T. forsythia)* in women not taking exogenous sex steroid hormones (those found in hormonal contraceptives) (Clark and Soory, 2006). The presence of *P gingivalis* has been associated with gingival inflammation during menstruation and pregnancy and positively correlated with the increase in sex hormones in saliva (Muramatsu and Takaesu, 1994; Carrillo-de-Albornoz et al., 2010). In addition to the red complex bacteria, the closely related *P. endodontalis* also proved to be significantly correlated with increasing estradiol (Lombardo Bedran et al., 2012). Although estradiol has been reported to exert both pro- or anti- inflammatory responses in oral mucosa or modify growth of specific oral species at a dose dependent manner, yet the mechanisms by which this occurs have not been explored in depth. We also observed that the abundancies of *Prevotella* and *Veillonella* were increased in current smokers, as also demonstrated earlier (Kumar, 2013; Paropkari et al., 2016). These changes may not be surprising as smoking leads to a diverse, pathogen-rich anaerobic oral microbiome and depletion of commensals, hence creating an at-risk-for-harm environment for the development of oral diseases (Shchipkova et al., 2010). This additive risk could may well apply to the female population, where hormonal regulations are concurrent with the transient establishment of a dysbiotic microbiota (Paropkari et al., 2016).

While there is a circumstantial body of evidence linking the menstrual cycle to changes in the microbiome of the dental plaque, there is no clear consensus and the sample size of the cohorts were rather limited. An association between ovulation and the increased levels of anaerobic bacterial counts in saliva has been supported (Prout and Hopps, 1970) whereas others did not conclude on a cyclical pattern of subgingival bacterial colonization of any of the 74 species studied (Fischer et al., 2008). Yet, the study has described that *Aggregatibacter actinomycetemcomitans*, a species closely related to the *Haemophilus* genus, was commonly detected at the beginning of the menstruation and peaked during the following 2 weeks. While *A. actinomycetemcomitans* itself was not frequently identified in the present study, *Haemophilus* was among the four differentially abundant genera through the cycle, represented by species other than *A. actinomycetemcomitans*. *Campylobacter rectus (C. rectus*) is a gram‐negative motile rod associated with periodontal diseases, whose growth can be enhanced by estradiol (Yokoyama et al., 2005; Bostanci et al., 2007; Yokoyama et al., 2008). Young pregnant or postpartum women carry high levels of *C. rectus* in subgingival plaques or saliva (Mitchell-Lewis et al., 2001; Yokoyama et al., 2005). A sharp rise in salivary estradiol levels is shown to occur immediately at the ovulation phase (Lu et al., 1999), and such hormonal peaks may indeed explain the significant fluctuation in the abundances of *Campylobacter* and *Prevotella* genera during the progression of the menstrual cycle observed in the present study. While there are at present no reports linking the presence or growth of *Oribacterium* to progesterone and estradiol, it is interesting to note that the occurrence of this genus has been associated with oral malodor, or halitosis (Sizova et al., 2014; Seerangaiyan et al., 2017). The menstrual cycle has also been pointed out as a factor influencing halitosis. Distinct cyclic variations in volatile sulphur compound concentrations occur during the menstrual cycle, which seem to coincide with the mid-cycle surge of the luteinizing hormone and the mid-luteal phase, corresponding to a peak of progesterone and estrogens, respectively (Tonzetich et al., 1978; Kawamoto et al., 2010).

The present study did not identify any major influences of the intake of oral contraceptives in the composition of the salivary microbiome composition. This is further supported by earlier studies demonstrating that the magnitude of gingival inflammation is not affected by various oral contraceptive formulations (Preshaw et al., 2001). Yet, improved oral hygiene may compensate for the potential hormonal influence on the oral microbiome due to oral contraceptive intake (Preshaw et al., 2001; Preshaw and Bissett, 2013). *Rothia, Haemophilus*, and *Neisseria* were highly abundant among the samples. These genera are known to be associated with good oral health, a finding that is aligned with the data obtained from the questionnaires (Palmer et al., 2017). A few early studies attempted to shed light on potential relationships between oral contraceptives and the oral microbiota, demonstrating a higher percentage of *Bacteroides* spp., such as *Prevotella intermedia* (previously *B. intermedius*), in women under a hormonal contraception regimen (Jensen et al., 1981).

It is becoming increasingly apparent that determining the structural composition of the oral microbiome delivers a finite amount of information, and that evaluating its function is crucial to understanding its totality (Espinoza et al., 2018). The present study identified selective functional variations of the salivary microbiome during menstrual phases, revealing its adaptability to hormonal fluctuations, while the overall community remains structurally intact. The most prevalent microbial functional modules were highly consistent within and across subjects. Yet, a subset of functions significantly differed between cycle phases, but also between contraceptives users. Interestingly, we found that the capacity for the biosynthesis of GABA was decreased during the menstrual phase. GABA is a neurotransmitter of major importance to inhibit functionality in the brain. GABAergic deficits are suggested to contribute to poor mental health and reduced GABA levels have been found in depressed patients, both centrally and in the periphery. Premenstrual dysphoric disorder has been linked to alterations in systems related to GABA (AC et al., 2006; Hofmeister and Bodden, 2016), however most significantly shown in the luteal phase. Notably, there are also previous reports on elevated inflammation as well as subjective health symptoms to be present in a higher level during the menstrual phase, also in women with no premenstrual syndrome (Puder et al., 2006). Smoking and sugar consumption seem to associated with differences in metabolic profile of the salivary microbiome. Most significantly associated with smoking was the pyrimidine deoxyribonucleotide biosynthesis, which was evidently higher in smokers, and high sugar consumption, which was associated with reduced phosphatidylethanolamine biosynthesis. It is interesting to note that pentose phosphate metabolism is frequently increased in Gram-positive pathogens such as *Streptococcus* and *Actinomyces* in response to environmental stresses. In the present data set, gram positive bacteria such as *S. mitis* and *S. oralis* were reduced in abundance during the luteal phase that had low gene abundance in the pentose phosphate pathway. On the contrary, genes related to membrane transport including the phosphotransferase system, RTX-toxin transport, RaxAB-RaxC type I secretion were over-represented during the luteal phase, which may be related to bacterial chemotaxis and increased toxin biosynthesis. Although the differences in gene richness did not seem to correspond to differences in bacterial species richness, this is in line with an earlier study indicating that 50% of all genes in a metagenomic sample are individual-specific and the functional differences between individuals are larger than the taxonomic differences (Tierney et al., 2019). A number of species within the oral microbiome community can metabolically exploit hormones as carbon and energy sources, processing them by degradation or chemical modification (Takahashi et al., 2010; Garcia-Gomez et al., 2013; Kumar, 2013).

In conclusion, we longitudinally characterized the ecological shifts associated with hormonal fluctuations in the salivary microbiome of regularly menstruating women, during the course of one full menstrual cycle. This is the first large study to systematically address dysbiotic variations of the oral microbiome during the course of menstrual cycle, and document the additive effect of smoking and sugar consumption as environmental risk factors. It reveals the structural resilience and functional adaptability of the oral microbiome to the endogenous hormonal pressures of the menstrual cycle, while revealing its vulnerability to the exogenous exposures of diet and smoking.

## Data Availability Statement

All sequencing data analysed in this study are available from the European Nucleotide Archive under project PRJEB37731, samples SAMEA6662389-SAMEA6662857.

## Ethics Statement

The participants gave both oral and written consent to participate in this study.

## Author Contributions

MK, ZB, and HN obtained ethics and data protection approval, wrote the procotol, planned and organized the study cohort, included participants and secured informed consent and collected samples. LH, NB, FB, and GB analyzed the sequencing data and wrote the manuscript. IS-K planned experiments and wrote the manuscript. EF and LE wrote the manuscript and supervised students. KW contributed to conception and design of the manuscript. All authors contributed to the article and approved the submitted version.

## Funding

The Centre for Translational Microbiome Research is partly funded by Ferring Pharmaceuticals (LH, FB, LE, IS-K, and EF). A research grant from Ferring Pharmaceuticals enabled the clinical recruitment and sampling (HN). The funder was not involved in the study design, collection, analysis, interpretation of data, the writing of this article or the decision to submit it for publication. The Rigshospitalet Research Fund (HN and MK), Karolinska Institute Strategic Funds (NB and GB), the Swedish Research Council (NB), and KI/SLL Strategic Dental Research Fund (NB and GB).

## Conflict of Interest

Author KW was employed by Ferring International Center SA.

The remaining authors declare that the research was conducted in the absence of any commercial or financial relationships that could be construed as a potential conflict of interest.
