# Supplementary material for: Dysbiosis of the Human Oral Microbiome During the Menstrual Cycle and Vulnerability to the External Exposures of Smoking and Dietary Sugar
Source: Front Cell Infect Microbiol. 2021 Mar 19;11:625229. doi: 10.3389/fcimb.2021.625229 (PMC8018275; doi:10.3389/fcimb.2021.625229)
Supplement: Supplementary file 1 [file DataSheet_1.pdf]

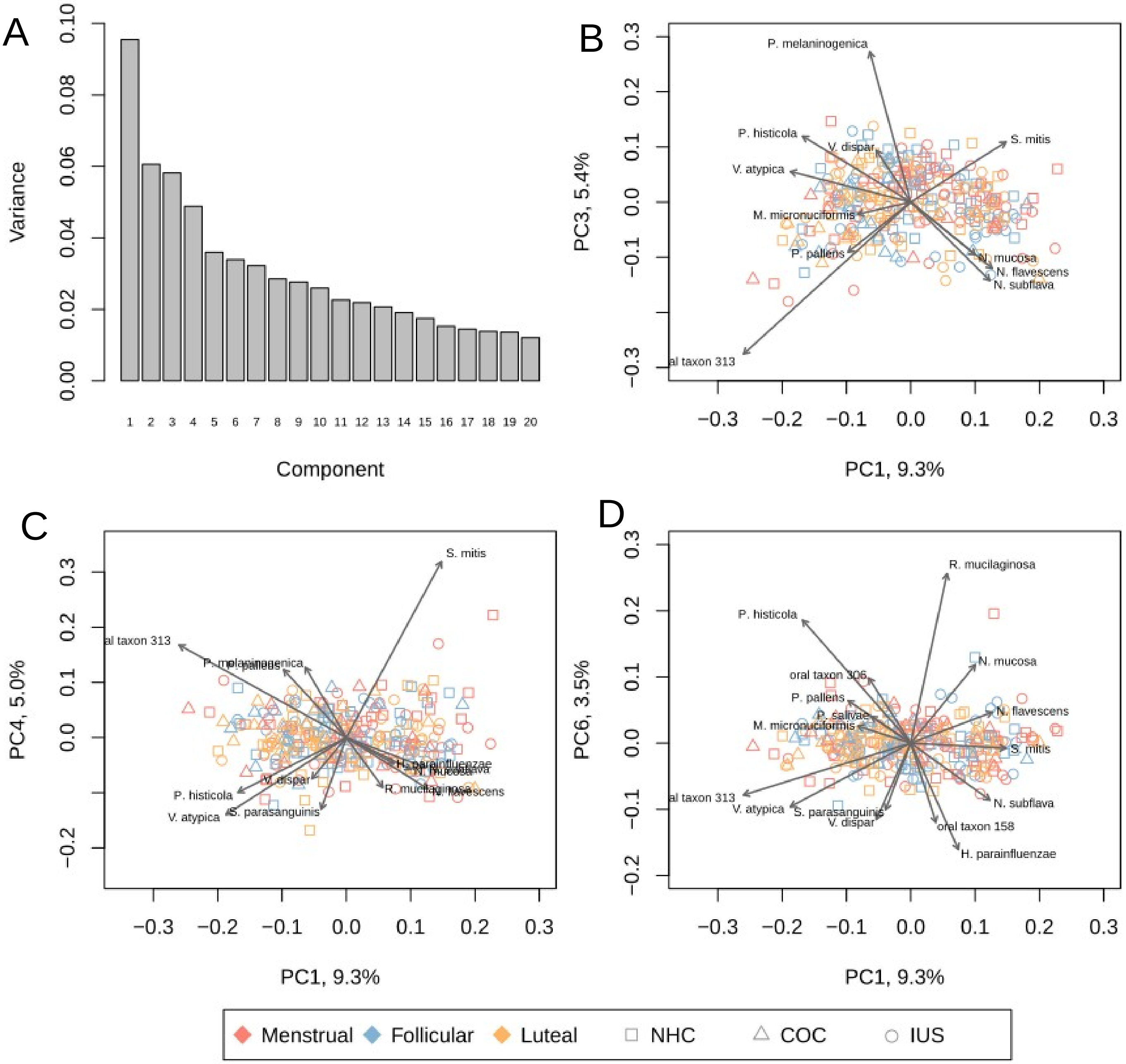

Figure S1: A) Variance explained by each of the first 20 principal components. **(B-D)** PC2, PC3 and PC6, respectively, plotted against PC1.The species with highest impact on the depicted principal components are overlaid as gray arrows. Red: menstrual phase. Yellow: follicular phase. Blue: luteal phase. Circle: non-hormonal contraceptives. Square: combined oral contraceptives. Triangle: intra-uterine levonegestrel system. *H. parainfluenzae: Haemophilus parainfluenzae; N. flavescens: Neisseria flavescens; N. mucosa: Neisseria mucosa; N. subflava: Neisseria subflava; N. sicca: Neisseria sicca; oral taxon 306: Prevotella sp. oral taxon 306; oral taxon 313: Prevotella sp. oral taxon 313; P. histicola: Prevotella histicola; P. melaninogenica: Prevotella melaninogenica; P. pallens: Prevotella pallens; R. mucilaginosa: Rothia mucilaginosa; S. mitis: Streptococcus mitis; S. parasanguinis: Streptococcus parasanguinis; V. atypica: Veillonella atypica; V. dispar: Veillonella dispar*

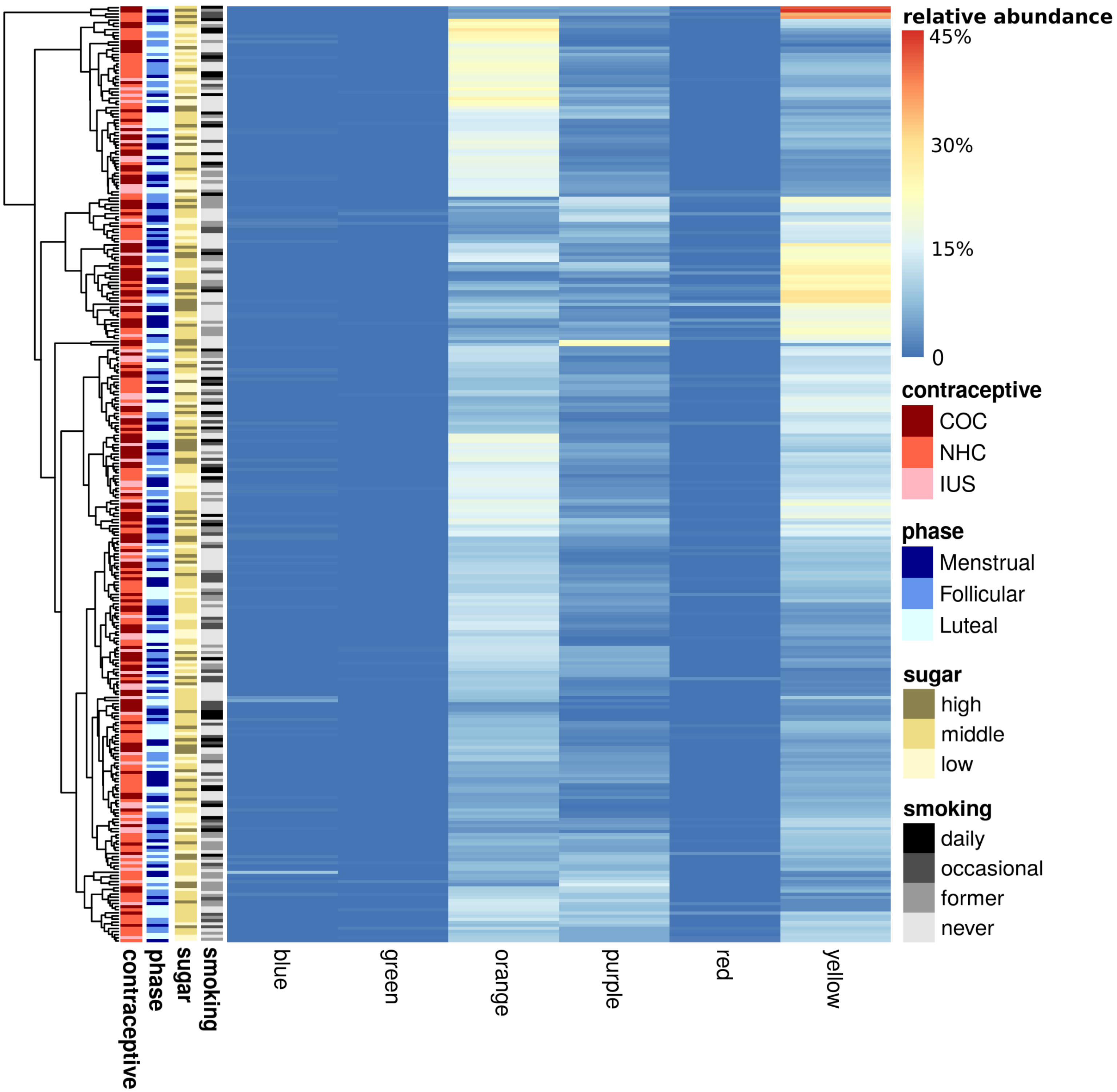

Figure S2: Heatmaps presenting relative abundance for for species of the Socransky´s complexes. The blue, yellow, green, and purple complexes are associated with periodontal health, whereas the orange and red complexes are correlated with periodontal disease. The four columns on the left show the contraceptive usage, menstrual phase, sugar consumption and smoking status associated to each sample.

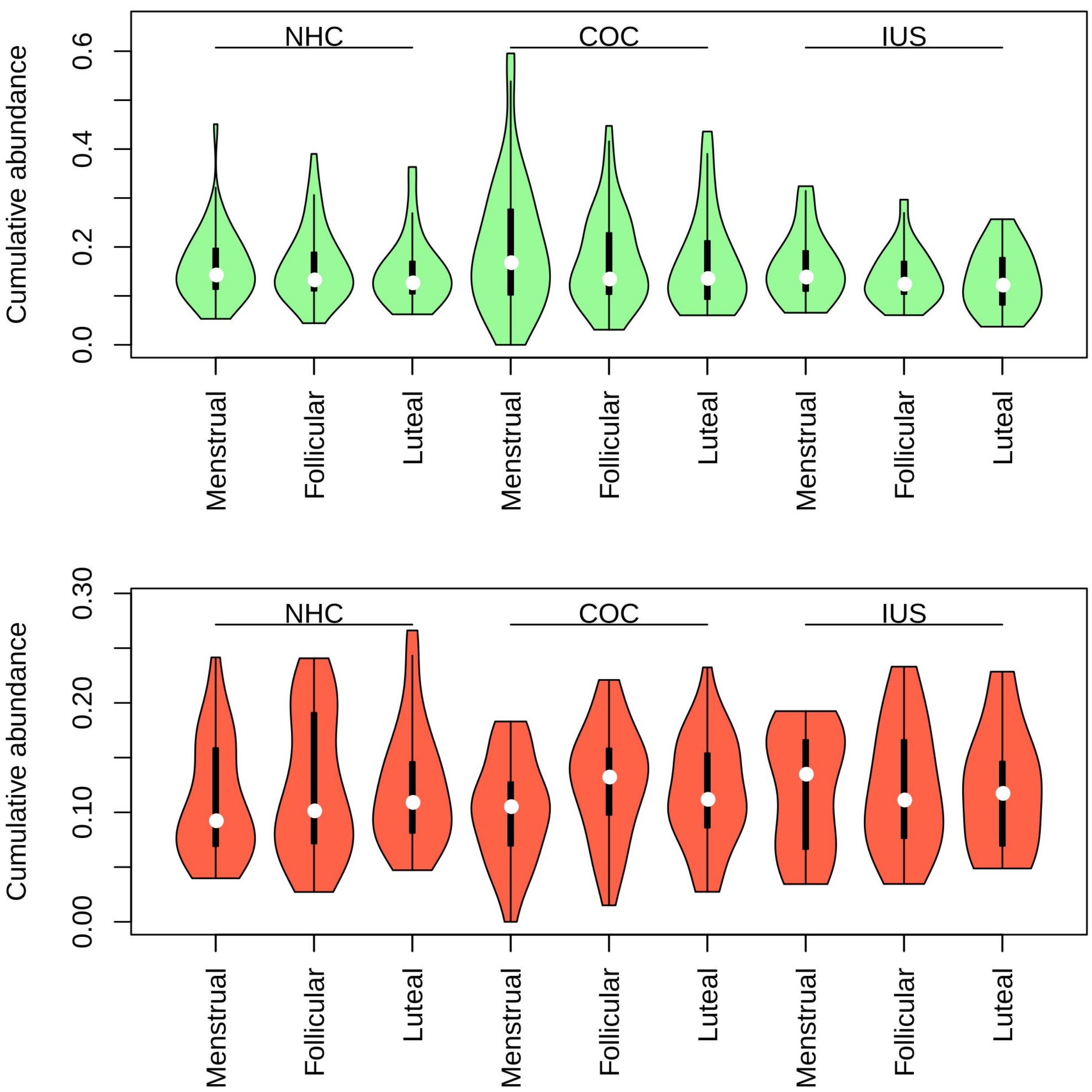

Figure S3: Violin plot representing the distribution in relative abundance for each phase of the menstrual cycle and contraceptive method according to the core microbiome classification data .
green: health-associated; red: periodontal disease-associated

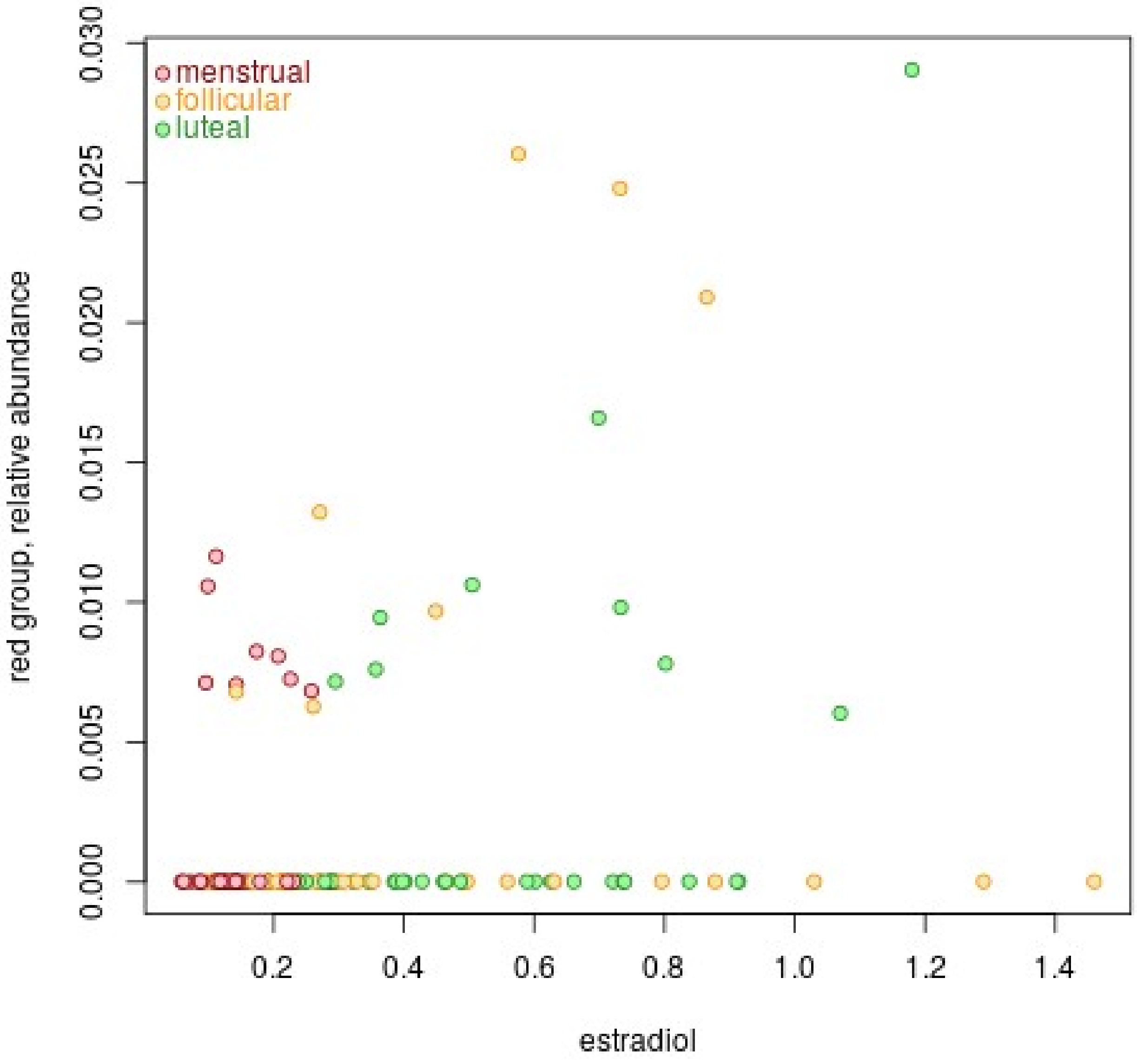

Figure S4: Scatter plot depicting the abundance of red group bacteria in relation to plasma estradiol concentrations for women not using hormonal contraception.
Dots are colored after the phase in the menstrual cycle: red, menstrual; yellow, follicular; green, luteal.
